# Supplementary material for: Identification and characterization of functional modules reflecting transcriptome transition during human neuron maturation
Source: BMC Genomics. 2018 Apr 17;19:262. doi: 10.1186/s12864-018-4649-2 (PMC5905132; doi:10.1186/s12864-018-4649-2)
Supplement: Supplementary file 1 — Figure S1. Robustness of module identification to the choice of insulating parameter (β). (DOCX 213 kb) [file 12864_2018_4649_MOESM1_ESM.docx]

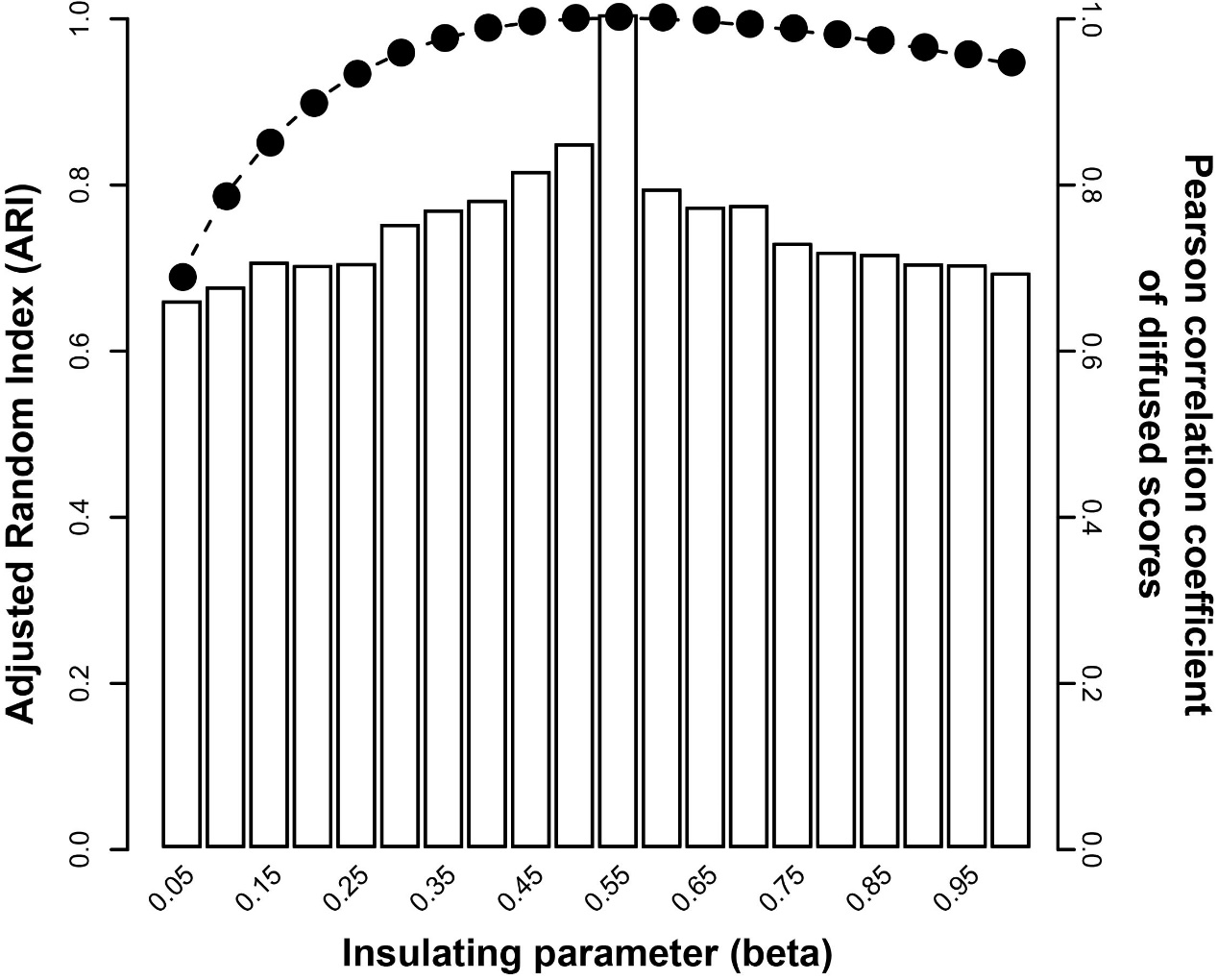


**Figure S1**. Robustness of module identification to the choice of insulating parameter (β). The x-axis shows 20 different choices of β ranging from 0.05 to 1 with step size of 0.05. Height of each bar shows the adjusted random index (ARI) between modules identified with β=0.55 and those with β set to be the corresponding value as shown by the x-axis. ARI calculates the proportion of agreements between two groupings with adjustment to random performance. Shadow bars show the proportions comparing to random modules. Dots show Pearson correlation coefficient of expression alteration scores after diffusion between choices of β being 0.55 and the value shown by the x-axis.
