# Supplementary material for: Identification and characterization of functional modules reflecting transcriptome transition during human neuron maturation
Source: BMC Genomics. 2018 Apr 17;19:262. doi: 10.1186/s12864-018-4649-2 (PMC5905132; doi:10.1186/s12864-018-4649-2)
Supplement: Supplementary file 4 — Figure S2. Modular and integrated NMIs of samples in Darmanis et al... dataset. (DOCX 735 kb) [file 12864_2018_4649_MOESM4_ESM.docx]

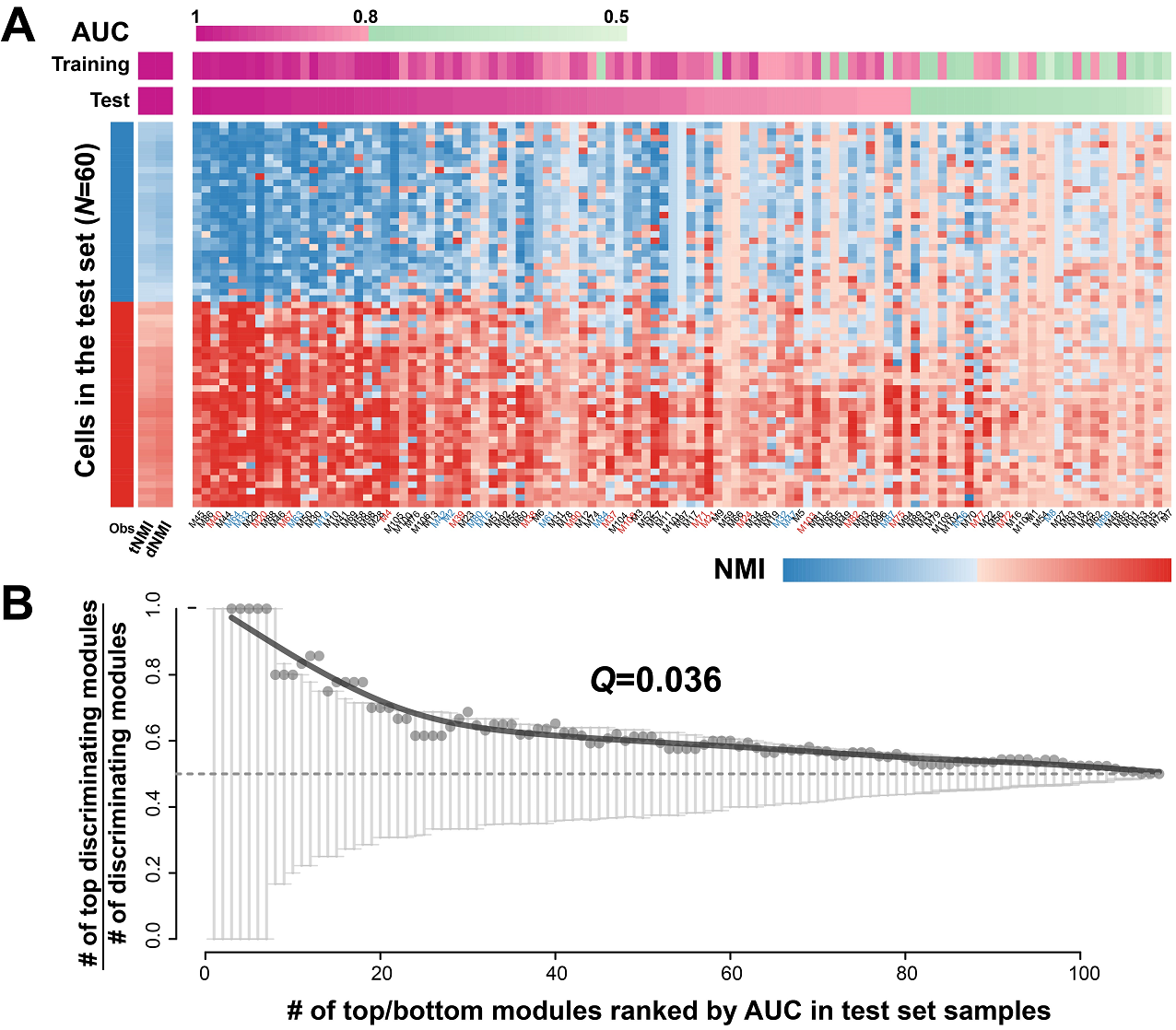


**Figure S2**. Modular and integrated NMIs of samples in Darmanis et al. dataset. (A) Performance of NMI to estimate neuron maturity state. Bars on top show performance of tNMI, dNMI and each of the mNMIs in prediction of neuron maturity state using Darmanis et al. dataset, as indicated by Area under curve (AUC) of Receiver operating characteristic (ROC). AUC of the training set is calculated based on ten-fold cross-validations. The heatmap shows the estimated NMIs for each neuron in the test set, with each column represent one of tNMI, dNMI and mNMIs of discriminating modules. Module labels are colored based on expression changes of genes in the modules during neuron maturation: red – higher in mature neurons, blue – higher in immature neurons. The real neuron maturity states are shown by the every left column: red – mature neurons, blue – immature neurons. (B) NMIs of discriminating modules perform better than other mNMIs. Y-axis shows ratio between the number of discriminating modules among the top-*N* modules ranked by their mNMI performance in the test set, to the number of discriminating modules among the top-and-bottom-*N* (in total 2*N*) modules. X-axis shows variable *N*. Dots show the observed ratios, with the curve showing the smoothen pattern (natural spline, *df*=5). Grey arrows show the 90% confident intervals based on 1000 permutations of module ranks.
