# Supplementary material for: Identification and characterization of functional modules reflecting transcriptome transition during human neuron maturation
Source: BMC Genomics. 2018 Apr 17;19:262. doi: 10.1186/s12864-018-4649-2 (PMC5905132; doi:10.1186/s12864-018-4649-2)
Supplement: Supplementary file 5 — Figure S3. Applications of tNMI in human brain single cell RNA-seq data of neurons to investigate neuron maturity dynamics. (DOCX 154 kb) [file 12864_2018_4649_MOESM5_ESM.docx]

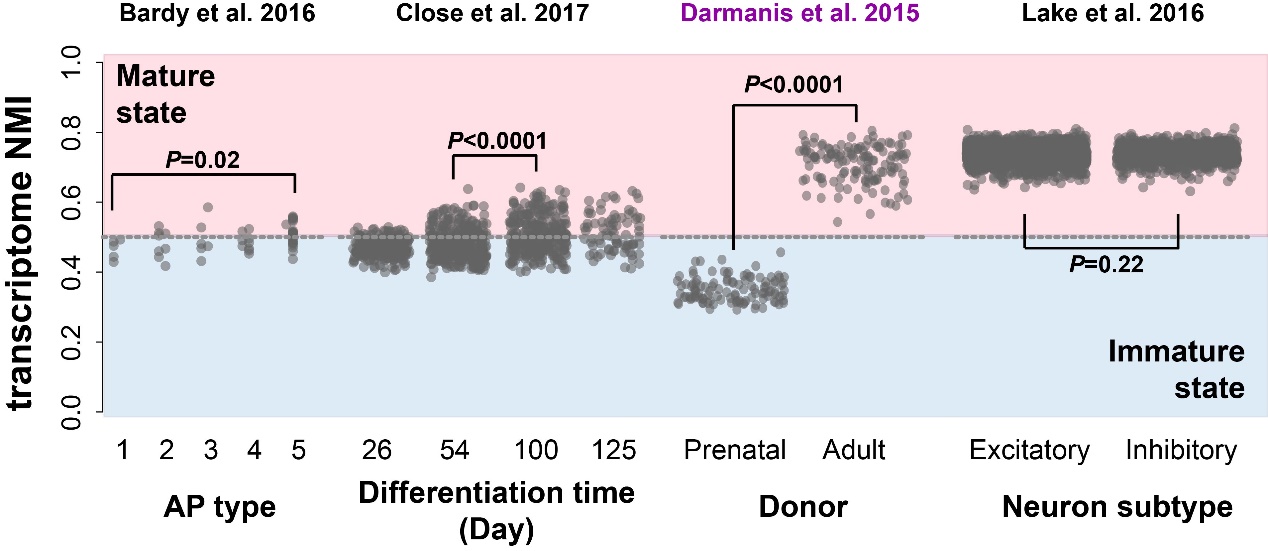


**Figure S3**. Applications of tNMI in human brain single cell RNA-seq data of neurons to investigate neuron maturity dynamics. It shows the estimated tNMI of each neuron sample, as represented by the y-axis, in four public single cell/nucleus RNA-seq data sets. Each dot represents one cell. The dash line represents NMI=0.5 as the boundary of estimated immature and mature state. For each of the four data sets, cells are grouped based on the respective metadata: Bardy et al. 2016 dataset: action potential (AP) type; Close et al. 2017 dataset: differentiation time; Darmanis et al. 2015: cell donor ages; Lake et al. 2016: neuron subtypes (excitatory and inhibitory neurons). P values of Wilcoxon rank sum test are shown for comparisons of dNMIs between neuron subgroups in each dataset. Purple label on top marks the dataset used to train the NMI model (Darmanis et al. 2015 dataset).
