# Supplementary material for: Identification and characterization of functional modules reflecting transcriptome transition during human neuron maturation
Source: BMC Genomics. 2018 Apr 17;19:262. doi: 10.1186/s12864-018-4649-2 (PMC5905132; doi:10.1186/s12864-018-4649-2)
Supplement: Supplementary file 6 — Figure S4. Transcriptome signatures of single neurons are driven by maturity states rather than batch effect across datasets. (DOCX 185 kb) [file 12864_2018_4649_MOESM6_ESM.docx]

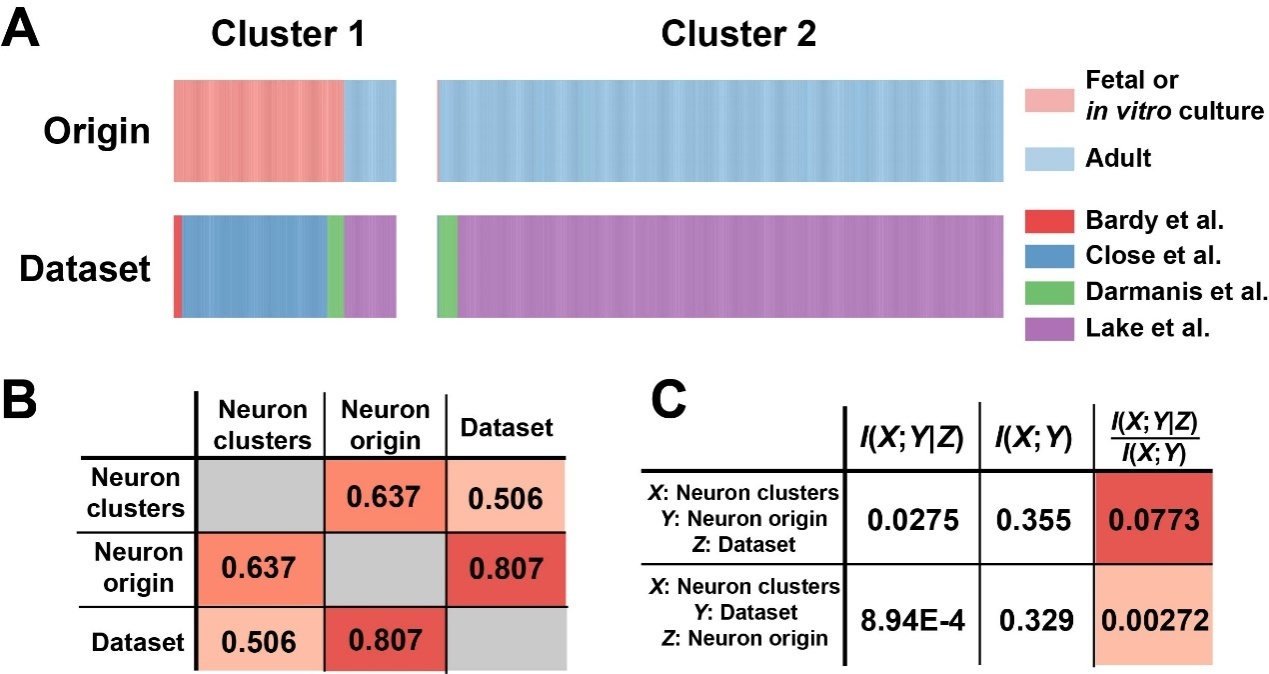


**Figure S4**. Transcriptome signatures of single neurons are driven by maturity states rather than batch effect across datasets. (A) Origins and datasets of single neurons in the two neuron clusters identified by hierarchical clustering based on standardized expression levels of signature genes for NMI estimations. The two columns show neurons grouped in the two neuron clusters, with the two rows show their origins (red – from fetal brains or *in vitro* cultures) and datasets (red – Bardy et al. dataset; blue – Close et al. dataset; green – Darmanis et al. dataset; purple – Lake et al. dataset). (B) Normalized pairwise mutual information among the neuron clusters, neuron origins and datasets across different single neurons. Darkness of red shows the strength of dependency. (C) Conditional mutual information between neuron clusters and either neuron origins or datasets, under the condition of the other one. The first two columns show the corresponding conditional mutual information and mutual information, with the third column showing the ratio of the first two columns. Darkness of red shows proportions of conditional mutual information among mutual information.
